# An Argument Against Cross-Platform Development: Lessons From an Augmented Reality App Prototype for Rural Emergency Responders

**DOI:** 10.2196/12207

**Published:** 2019-03-28

**Authors:** Bryan Weichelt, Tomi Heimonen, Matthew Pilz, Aaron Yoder, Casper Bendixsen

**Affiliations:** 1 Marshfield Clinic Research Institute National Farm Medicine Center Marshfield, WI United States; 2 University of Wisconsin-Stevens Point Stevens Point, WI United States; 3 linkedPIXEL LLC Marshfield, WI United States; 4 Department of Environmental, Agricultural and Occupational Health University of Nebraska Medical Center University of Nebraska-Lincoln Omaha, NE United States

**Keywords:** rural health, mhealth, telemedicine, emergency medical services

## Abstract

**Background:**

Mobile augmented reality (MAR) apps offer potential support for emergency responders in rural areas.

**Objective:**

In this report, we described lessons learned from the development process of augmented reality (AR) Farm Mapping to Assist, Protect and Prepare Emergency Responders (MAPPER), a MAR app that provides emergency responders onsite information about the agricultural operation they enter.

**Methods:**

Cross-platform frameworks were used to create AR MAPPER to accommodate budget constraints and overcome issues with markerless MAR technologies. Although the single codebase and Web technologies streamlined development, cross-device hardware limitations impacted location accuracy, lengthened the development cycle, and required regular updates to third-party libraries.

**Results:**

A hybrid development approach of using Web-based technologies with native tie-ins for specialized components and enhanced performance cut time and costs. This also led to consistency across multiple platforms and ensured that there is only a single set of source files to modify for Android and iPhone operating systems. Meanwhile, active development was delayed by some major hurdles. Apple and Google both released new versions of their operating systems, and the Wikitude framework issued four major updates, each of which brought with it some important enhancements and also led to some new issues.

**Conclusions:**

Developers should consider single platform native development to benefit from platform-specific MAR implementations and to avoid development, testing, and maintenance costs associated with cross-platform implementation. Emergency response organizations may be more likely to utilize a single platform across the devices used by their command staff. This also reduces the benefits of cross-platform development. Furthermore, providing map-based, non-AR cross-platform apps for landowners, farmers, and ranchers would help improve and maintain data quality, which is crucial for the utility and user experience of MAR apps.

## Introduction

Augmented reality (AR) [1] combines a view of the real world with digitally overlaid content. Recently, mobile augmented reality (MAR) apps have become increasingly popular, where virtual objects are combined with objects in the real environment in real time and aligned based on the user’s point of view through their mobile device display [2]. MAR games and improving support from mobile platform providers have made MAR accessible to end users and developers. However, users have several expectations toward MAR apps and that the underlying technology will influence the user experience (UX). MAR interfaces are expected to be able to provide valid, up-to-date, and relevant content to the user [3]. This may be particularly true in the context of apps created for medical education, health care service delivery, and other industries where unreliable and irrelevant content could have material and safety implications. Given these requirements, decisions made early in development have a significant effect on whether MAR apps provide reliable and relevant content. Developing a modern and cross-platform MAR app can easily accumulate significant expenses because of the sheer magnitude of resources required [4]. In practical terms, a key decision is whether to develop native apps for 1 or multiple mobile platforms or to use a cross-platform approach whereby hybrid app development frameworks are utilized to deliver the app to multiple platforms.

Farm Mapping to Assist, Protect and Prepare Emergency Responders (MAPPER) provides emergency responders onsite information about the agricultural operation they are entering [5]. This case report describes development lessons learned in creating the AR Farm MAPPER, specifically addressing cross-platform development. The MAR prototype of Farm MAPPER improves on the earlier, static overhead version by incorporating a real-time depiction of icons, such as hazards, resources, and points of entry. This offers the possibility for on-scene commanders to have a heads-up display. This report, which builds upon a previously published manuscript [6], may be of more interest to researchers and practitioners working with MAR in the medical informatics field and, particularly, when considering issues related to the choice of approach—cross-platform development versus targeting single platforms. The specifics of the original Farm MAPPER app and its applicability to agricultural injury prevention interventions are the topic of another paper [6].

## Methods

The project was led by a research team, which was administratively housed within a private rural health care system in the Upper Midwest. The skill set needed to develop and test MAR technology can be difficult to find. No internal resources were available. Thus, the lead developer (third author) recruited for this project was self-employed and subcontracted, although residing in the same community as the research team. The lead developer had significant experience in Web and mobile app development, with some familiarity with AR. The developer’s portfolio included first place awards for back-to-back public app development competitions run by Intel, the only developer to have done so. Few stakeholders were involved in this prototype’s development, which did not include formal usability testing.

The benefit of a Web-based approach is the ability to leverage any of the thousands of freely available libraries and extensions, many of which are licensed under MIT, Apache, or GNU, which makes them fully available for distribution in any type of app, free or commercial. This can save tens of thousands of work hours to reinvent many already existing components. Moreover, the complexity of managing multiple software development kits with their differing application programming interfaces (APIs), build systems, and tools can be avoided [7]. This approach, as opposed to developing fully native apps for each platform, represents a more efficient way to deploy a product that will work on most modern mobile platforms. This can be particularly useful if the app is expected to be used by multiple organizations, some of which may have already committed to a specific platform.

Although the window of time between our first written proposal and the final completion and delivery of the product extended more than a year, the fixed development budget only accommodated approximately 292 hours of actual labor (roughly equivalent to 7 full-time work weeks by a single person). Although remaining conscientious of the available budget and the relatively uncharted nature of markerless AR, we had to make some development decisions early on to effectively accommodate the various requirements. This led to the use of a third-party framework to simplify the AR mathematics and presentation as well as the use of Web-based technologies to retain a single universal codebase.

## Results

### What Went Right

It appears that taking a hybrid app approach toward development by using Web-based technologies (with native tie-ins for specialized components and enhanced performance) was a decision that had some benefits. This strategy helped ensure consistency across multiple platforms and ensured that there is only a single set of source files to modify for Android and iPhone operating system (iOS, Apple Inc) combined. We were able to also borrow from various user interface (UI) libraries, notably OnsenUI [8] and the Wikitude [9] frameworks, to expedite the implementation of key features while ensuring a consistent UX across both Android and iOS devices. The key benefits of Wikitude are the ability to program AR content using basic Web technologies and easy porting of apps between platforms [10].

Traditional development would have required use of native UI elements for all facets of the app; any changes made on one platform would then have to be manually worked into the source of the other platform. Requiring constant synchronization of 2 native apps would have effectively reduced the available work time by half. Conversely, the use of an existing and long-standing AR framework that supported hybrid apps within a universal API represented a substantial savings of time. Despite only an evaluation copy of the Wikitude framework being used throughout development and the considerable work that was still required to satisfactorily integrate it into the overall app, it offered a positive demonstration of the current capabilities and technical limitations of developing location-based AR.

### What Went Wrong

#### Physical Hardware Limitations

Going into this project, we knew that one of the largest setbacks we would encounter related to the technical limitations of modern-day hardware, particularly geolocation and global positioning system (GPS) accuracy. During testing across various devices, it was not uncommon for the variance in GPS accuracy to range anywhere from 30+ feet all the way up to 1400+ feet. This poses a problem when the objective of the app is to allow emergency responders the ability to quickly locate points of interest on a property. The nature of GPS also tends to require a delay between the initial GPS probe and an accurate fix. Although most modern cellular phones can typically lock onto GPS quite rapidly using assisted GPS technology, some devices we tested, including the LG G2, could take up to 10 min. Certain modern Android devices also lacked gyroscope functionality, which caused the AR framework to fail even though it was meant to be a universal solution. This then required additional correspondence with the framework developers who had to try and implement a workaround specifically for the affected devices.

#### Lapse in Timeline of Development

Despite a relatively short active development time (comparable with 1-2 months of dedicated work), the prolonged overall timeline of this project adversely impacted numerous aspects of it. There were multiple extended gaps between development from January to September, due in part to external occurrences, contractual matters, and other commitments. Although the generous timespan seemed appealing to better accommodate availability, it also led to struggles in having to make continual updates to the various frameworks and then retest all aspects to ensure no newfound issues emerged when subsequent operating system (OS) versions and libraries were released.

Within the project’s timeframe, Apple and Google both released sweeping new versions of their OS (iOS 11.0 and Android 8.0, respectively). Meanwhile, the Wikitude framework issued 4 major updates (from 6.0.1 to 7.1), each of which brought with it some important enhancements and also led to some new issues.

Given the basic prototype goals of this project, it would have been reasonable to develop and deploy the baseline product within a couple of months of dedicated work compared with stretching it over the course of a year and having to regularly update and recheck major components as the technologies evolved. A more focused, short-term deadline could have alleviated some of these obstacles.

## Discussion

### Arguments Against Cross-Platform Development

Cross-platform development is a popular buzzword. In many instances, it is indeed beneficial to accommodate all major platforms—currently Android and iOS. However, that is not always the case, and in retrospect, we would propose that this prototype could have benefited more directly if it had been built only for a single target platform. Organizations that provide work-appointed devices almost always adhere to a single platform strategy, such as a health clinic’s laptop distribution. This way, they only need to be concerned that the apps being used function as intended on the single platform and OS that they support.

The intent of this MAR project was to create a conceptual example of what emergency responders could use to locate areas of interest on a rural or agricultural property through visual cues such as hovering AR icons representing hazards, resources, and items of interest. Rather than relying on the emergency staff’s own personal phones for such activities, it is more probable that the facilities would supply identical, company-owned units to all personnel or at least individual shift leaders. In this respect, focusing on a single platform with a limited set of technology-related variables could help provide a more consistent UX.

In related research, a survey of the Google Play Store revealed that hybrid development is more popular as an approach to creating data-intensive apps and correspondingly less popular in apps that require closer interaction with the underlying OS [11]. App reviews show that native apps are generally perceived as more performant and less buggy (ibid.), and hybrid apps on iOS and Android were more prone to complaints in user reviews [12]. Mobile app developers may also favor native development, particularly when better UX and access to device-specific features were needed [13]. Several case studies have shown that although a reasonable UX can be achieved by using a cross-platform framework, there are still limitations with performance, UX, and usability [14-16].

### Benefits of Developing for iPhone Operating System Only

With the consideration to develop natively for a single platform, iOS would be an ideal choice if it is to be used as an internal company app. Although Android is equally capable and more versatile in many ways, from a development and industrial perspective, Android also has a vastly more diverse blend of devices, OS versions, and varying limitations that can impede development time and testing.

iOS also has a more streamlined adoption rate in which new OS versions are generally installed soon after being released (more than 95% of all iOS users are within 1 version of the latest OS) [17]. In comparison, Android users often must stick to older versions because of their carrier and device restrictions; so the adoption rate to newer versions is quite low (less than 40% of all Android users are within 1 version of the latest OS) [18].

**Table 1:** Potential app features.

Feature	Brief description
Caching and prefetching of map data	Currently, the top-down map imagery first begins downloading when the map view is brought into focus and is based on the user’s current location. For situations in which network data connectivity may be sparse, caching of data would prove valuable so that the location imagery and marker data can be fetched while somewhere with a strong connection instead of after arriving onto the site. With such functionality, the map could immediately download the image data of all quadrants surrounding the loaded markers for future access.
Sorting of markers by distance	The option to sort all markers by distance to user would allow the user to quickly review the closest resources when viewing the marker list view.
Enhanced GPS^a^ simulation	The current version includes rudimentary support for manually entering a static GPS location to simulate that vantage point. In a more robust integration, the simulated coordinates could be obtained automatically from the loaded location and allow the user to physically move within the simulated coordinates for remote practice and exploration.
Distance from one marker to another	It could prove beneficial to be able to quickly approximate the distance from one placed marker to another while reviewing the loaded data. For instance, to see how far a water source is to a barn.
Speech recognition and audio navigation	In cases where directly viewing and holding the phone may not be accommodating, being able to use speech (eg, “Find Water Source”) and have the app return audio cues on which direction to travel to find it could be helpful.

^a^GPS: Global Positioning System.

Finally, although Android OS spans thousands of different devices with a highly varying list of specs, iOS devices all use the same underlying architecture and are very limited in variety. An app developed for a modern version of iOS is almost certain to function identically on all the latest iPhones, with minimal design or developmental changes required to compensate for device-specific issues.

Perhaps most importantly, developing on only a single platform would allow the use of each platform’s respective AR framework. Apple shipped ARKit [19] alongside iOS 11 for the latest generation of iPhones, whereas Google debuted its competing system dubbed ARCore [20]. Neither of these frameworks are cross-platform, and they were not publicly available for much of this app’s lifecycle, but if developing a native AR app in the future, they should be highly considered. We anticipate that open-source libraries for hybrid apps will be developed to transparently support both AR toolsets depending on the platform, which will rival many features currently found in Wikitude.

### The Alternative iPhone Operating System–Only Approach—Heightened Focus on Functionality

Developing a custom AR component may have been more achievable had the focus been devoted entirely to a single platform. If so, this could have alleviated the need to depend on a costly and proprietary third-party commercial framework, which itself includes many excess features not needed or used by AR MAPPER.

During this project, a sizeable amount of time was spent troubleshooting and resolving device-specific issues while testing on Android. Even when using the popular hybrid approach, some native components still caused issues depending on the device. When Wikitude 7.0 was released, for example, it inadvertently broke the app on a couple of Android devices we used for testing that did not have certain sensors. This led to more time spent troubleshooting and ultimately having to point out the issues to the framework developers, who addressed the issue, but not formally for another 3 months.

### Other Potential Features for This Type of App

The time and budget for this project elapsed before several considered features could be properly assessed or integrated. A summary of such imagined features is described in Table 1.

### Evaluating Mobile Augmented Reality Apps

Although the proposed MAR app was not formally tested, several options exist to assess the success of mobile apps. Usability heuristics have been adapted to the design and evaluation of AR apps [21,22], but they are of somewhat limited utility when performance in the field is of interest. Success of AR app concepts can also be evaluated via surveys before design and development [23] or based on user reports on the use of an AR app [24]; however, these methods also suffer from lack of direct access to the real-world context of use. Although appropriate methods for field testing mobile apps are well understood [25], field evaluation of MAR has generally received less attention [26]. In situ, MAR evaluations have included various combinations of task-based assessment, observation, and subjective feedback collection through questionnaires and interviews [27-30]. Although such short-term, task-based evaluation can provide a baseline assessment of the immediate success of the MAR app, longitudinal studies would be more appropriate for uncovering issues that users would face in their day-to-day interactions.

This brief report is a snapshot in time in the development of a better, more useful farm mapping tool for emergency responders. As a part of a Centers for Disease Control and Prevention–funded research project, the original Farm MAPPER has been integrated as a tool for high-quality emergency planning for rural firefighters and the agricultural operations in their coverage areas. During trainings, these emergency responders are given a preview of the MAPPER AR and have been highly receptive. Of the 50 or so trainees, nearly all have voiced support for the heads-up style display that MAR technology allows. In future development, it is anticipated that valuable usability feedback will be easily attained, yielding meaningful and actionable results. Despite the limitations of the development caused by accommodating multiple platforms, the purpose of the new technology appears to be valued enough by the end users to allow for additional studies and builds.

Despite the rapid advancement and adoption of MAR, little is known about the effectiveness of these technologies in real-world rural emergency response [6]. Researchers in related disciplines, such as medical education, have also struggled to evaluate the technology’s place and effectiveness [31-36]. Next steps and future research must address the effectiveness and feasibility of this technology in the agricultural or occupational and patient care environments.

### Conclusions

From the early design and mockup phases to the final steps of development and testing, this project presented challenges. Many different frameworks and design methodologies were considered to satisfy the original scope and requirements while remaining within the budget. The result is a cross-platform, multi-view prototype app with aerial and AR components.

Undeniably, the biggest hurdle continues to be present-day hardware limitations, which cannot always guarantee a reliable connection to satellites or cellular towers required to parse data and track very granular and specific points of interest. This approach is not suitable for indoor navigation or other areas with heavy ground obstructions or tree foliage. An even greater concern exists in that many rural locations still do not have adequate cellphone reception. Although GPS signal is generally obtainable in these areas, the app still depends on network connectivity to download satellite imagery and initialize the various components correctly. Thankfully, the spread and improvement of carrier coverage and adoption of mobile devices continue to penetrate rural areas.

Developers should consider single platform native development to benefit from platform-specific MAR implementations and to avoid development and testing costs associated with cross-platform implementation. Emergency response organizations may be more likely to utilize a single platform across their devices, reducing the benefit of cross-platform development. Furthermore, providing map-based, non-AR cross-platform apps for landowners, farmers, and ranchers would help improve and maintain the data quality of AR content.

With the recently-launched Apple ARKit and Google’s ARCore framework and more broad concepts such as its Visual Positioning Service for indoor GPS tracking, we can assume that MAR will play increased roles in emergency response, health care delivery, and everyday life in the years to come. Enhancing everyday reality with accurate, meaningful, and visualized data can transform the training and execution of any number of skills, including emergency medical services and bedside health care. MAR technology can increase the amount of contextual data in emergency responses, thereby improving the decision-making capabilities of the users. In turn, this can expedite response times and protect responders. Combined, these elements may improve patient outcomes and increase other successes further downstream in health care delivery.

## Abbreviations

API: application programming interface
AR: augmented reality
GPS: Global Positioning System
iOS: iPhone operating system
MAPPER: Mapping to Assist, Protect and Prepare Emergency Responders
MAR: mobile augmented reality
OS: operating system
UI: user interface
UX: user experience
